# Liquid biopsies in primary and secondary bone cancers

**DOI:** 10.20517/cdr.2022.17

**Published:** 2022-06-21

**Authors:** Argia Ucci, Nadia Rucci, Marco Ponzetti

**Affiliations:** Department of Biotechnological and Applied Clinical Sciences, University of L’Aquila, L’Aquila 67100, Italy.

**Keywords:** Liquid biopsy, bone metastasis, osteosarcoma, Ewing sarcoma, extracellular vesicles, minimal residual disease, drug resistance

## Abstract

Liquid biopsies are a powerful tool to non-invasively analyze tumor phenotype and progression as well as drug resistance. In the bone oncology field, liquid biopsies would be particularly important to develop, since standard biopsies can be very painful, dangerous (e.g., when found in proximity to the spinal cord), and hard to collect. In this review, we explore the recent advances in liquid biopsies in both primary (osteosarcoma and Ewing sarcoma) and secondary bone cancers (breast, prostate, and lung cancer-induced bone metastases), presenting their current role and highlighting their unexpressed potential, as well as the barriers limiting their possible adoption, including costs, scalability, reproducibility, and isolation methods. We discuss the use of circulating tumor cells, cell-free circulating tumor DNA, and extracellular vesicles for the purpose of improving diagnosis, prognosis, evaluation of therapy resistance, and driving therapy decisions in both primary and secondary bone malignancies.

## INTRODUCTION

### Normal bone physiology: cellular players and the “virtuous cycle”

Bone is a connective tissue composed of a mineralized matrix and a cellular component. The former is made up of an inorganic part (mainly calcium phosphate) and an organic one, composed of type I collagen fibers as well as a plethora of proteins such as osteonectin, osteopontin, osteocalcin and proteoglycans. This bone matrix is also a storage for several growth factors belonging to the transforming growth factor (TGF)/insulin-like growth factor (IGF) superfamilies^[1,2]^. The two matrix components make bone a composite material with outstanding mechanical properties, but this comes with a caveat: bone needs to be modeled and remodeled during growth and adult life, respectively, otherwise its mechanical properties degrade over time, as is the case for elderly individuals of both genders and for some bone genetic diseases^[3,4]^. Proper bone modeling/remodeling is guaranteed by a finely tuned balance among the three main bone cells: osteoblasts, the bone-forming cells; osteoclasts, the bone-resorbing cells; and osteocytes, key controllers of bone matrix homeostasis^[5,6]^. During bone modeling, all bone sections change shape following both mechanical cues and microenvironmental factors and expand to accommodate the growing organism in physiological conditions. In adults, bone switches to remodeling, where osteoblasts and osteoclasts act on the same surfaces and constantly resorb and depose new bone, resulting in a net bone mass balance, following physiological mechanical stimuli as well as the organismal needs for calcium and phosphate^[7]^. Although this might appear to be a waste of energy, it is crucial to maintain proper mechanical features and guarantee the repair of microfractures that are detected by osteocytes, which subsequently drive the remodeling of the damaged surfaces. Osteocytes are also able to sense mechanical loading, which acts as an anabolic stimulus that promotes osteoblast activity^[8,9]^. Since the process of bone resorption-deposition in homeostatic conditions keeps bone at its best, and it is cyclic, bone researchers often use the term “virtuous cycle” to describe it.

### Primary bone tumors

Osteosarcoma is the most common primary bone tumor. It has two separate incidence peaks, the highest one between 10 and 20 years of age and a secondary one in people aged ≥ 65 years ^[10]^. Although the overall incidence of this cancer is low (3.1 cases per million per year in the US)^[11]^, it represents 2% of all cancers in 0-14-year-old and 3% in 14-19-year-old^[12]^, and its consequences can be devastating, ranging from limb amputation (20% of operable osteosarcomas) to death because of lung metastases^[11]^. Osteosarcoma derives from a malignant transformation of the mesenchymal cells that can present different cellular features, but they all have in common the deposition of osteoid-like matrix and mineralized or demineralized lesions that are clearly visible upon X-ray examination^[13]^. When the gold standard treatment, i.e., neo-adjuvant chemotherapy-surgery-adjuvant chemotherapy, is not possible, the only available treatment course is chemotherapy alone, since radiotherapy does not provide significant benefits and increases the risk of infection^[14]^. However, despite advances in the field, chemoresistance is a serious problem in this type of neoplasia, and the survival benefits of chemotherapy are limited due to the onset of drug resistance^[14-16]^, which eventually leads to lung metastasis development, inevitably ending with the death of the patient. Chemotherapy is therefore a limited tool in osteosarcoma management, but having a reliable way to monitor the onset of drug resistance would make it much more effective.

There is still no consensus on the cell from which Ewing sarcoma originates, but what is certain is that it most often localizes in bone (80%-85%) or the soft tissue surrounding it (15%-20%)^[17]^. Ewing sarcoma also has quite a high incidence of 2.93 per million in newborns to adolescents^[18]^. Moreover, 85% of Ewing sarcomas harbor a specific chromosomal translocation [t(11:22)(q24:q12)] leading to the fusion of the N-terminal portion of Ewing sarcoma gene (EWS) with the C-terminal portion of Friend leukemia integration 1 transcription factor (FLI1), which encodes for a chimeric protein that behaves as a transcription factor and modulates a plethora of cellular processes, eventually leading to malignant transformation^[17]^. While most other bone cancers have specific morphological features that make tracking their lineage relatively easy, Ewing sarcoma cells are round, small basophils and defined as “uniformly undifferentiated”^[17]^. Despite advances in the field of Ewing sarcoma treatment, 70% of metastatic patients, in whom lungs are most frequently affected as a secondary site, will eventually perish^[19]^. Treatment of this tumor is multimodal and comprises surgery, radiotherapy, and chemotherapy^[20]^. However, drug resistance is a very concrete issue, which sadly tends to increase after more aggressive rounds of chemotherapy that, in fact, do not seem to provide significant survival benefits and is especially marked in relapsing Ewing sarcoma^[21,22]^. Hence, monitoring the onset of drug resistance with a non-invasive technique such as liquid biopsy could be a valuable tool in the fight against this family of tumors as well.

### Bone metastases and the “vicious cycle”

The bone milieu is a particularly attractive microenvironment for metastasis development by many primary tumors, such as breast, prostate, lung, and kidney cancers^[23-25]^. There could be several reasons for this “preference”, such as the richness in growth factors of the bone matrix^[26]^, the presence of higher levels of calcium, which acts as a growth-promoting factor^[27,28]^ especially following bone resorption, and the particular structure of bone/bone marrow blood vessels, which are fenestrated, hence quite permissive for tumor cells extravasation. In fact, metastasis is a stepwise process, in which first cancer cells have to invade locally in the primary site, after gaining the expression of specific sets of molecules such as matrix metalloproteinases^[29,30]^, migrate into the bloodstream or lymphatic system, escape immune killing while circulating, extravasate in the secondary site, engraft, and finally survive in the secondary organ. The final parts of the process, spanning from the extravasation to the survival in the new microenvironment, are often termed “homing” and require cancer cells to “trick” the resident cells into believing that they actually belong there, expressing bone-specific factors (osteomimicry)^[31] ^or hematopoietic stem cell-specific factors (HSC-mimicry)^[32]^. 

Depending on the type of cancer, osteoblasts, osteoclasts, and osteocytes are affected in specific ways. In the case of breast cancer, metastatic dissemination usually causes the local degradation of the bone matrix, which is evident on X-ray analysis as void areas where bone should be. These types of metastases are termed “osteolytic”, and while both osteoblasts and osteoclasts are involved in their establishment, the main culprit for their radiographic appearance and fueling are osteoclasts, which become overactivated following their interaction with cancer cells^[33,34]^. On the other hand, prostate cancer preferentially causes a net activation of osteoblasts in the bone metastatic microenvironment, leading to abnormally high bone deposition and the onset of radio-dense spots identifiable by X-ray examination. These metastases are termed “osteosclerotic” or “osteoblastic”^[24]^. Both cancers can present osteolytic and osteosclerotic features in the same anatomical site, and in this case, bone metastases are defined as “mixed”^[23]^. The process leading to osteolytic or osteosclerotic lesions relies on a pathological cross-communication between cancer cells and bone cells, where the former tilt the balance towards bone resorption or bone deposition, hijacking the virtuous cycle of cross-regulation between osteoblasts and osteoclasts into an osteolytic or osteosclerotic “vicious cycle”.

In the former, cancer cells that reached the bone/bone marrow microenvironment secrete factors that induce a net increase in osteoclast differentiation and activity. These factors can act directly [e.g., tumor necrosis factor-α, interleukin (IL)-6, and IL-1β], and/or indirectly [like parathyroid hormone-related protein (PTHrP)], through the promotion of osteoblastic expression of pro-osteoclastogenic factors such as receptor activator of nuclear factor κB (RANKL) and macrophage colony-stimulating factor (M-CSF)^[26,33-35]^. The increase in osteoclast activity and differentiation leads to the degradation of bone matrix and the release of IGF-1, TGF-β, platelet-derived growth factor (PDGF), and bone morphogenetic proteins (BMPs) from it, which in turn stimulate tumor growth, thus creating a feed-forward loop, with bone cells, i.e., the osteolytic vicious cycle. In the osteosclerotic vicious cycle, cancer cells secrete a different set of proteins, including IGF-1, wingless-related integration site-1 (WNT-1) and WNT-3A, BMPs, and endothelin (ET)-1. These promote osteoblast differentiation and activity, leading to deposition of primary bone and production of growth factors^[24,36,37]^. Tumor growth is therefore fomented by osteoblasts, thus closing the osteosclerotic vicious cycle. Osteoblast differentiation also stimulates osteoclast differentiation, which further exacerbates the release of matrix-bound growth factors, with dangerous and painful consequences^[23,37]^.

## LIQUID BIOPSY IN BONE ONCOLOGY

### Liquid biopsies: what, how and why

Liquid biopsies are obtained in body fluids that are in direct contact with cancerous tissue and can provide important insights into the tumor without having to remove a part of it directly^[38,39]^. This is especially important for those tumors that are not easily accessible and for which even drawing a simple biopsy core may be dangerous and/or extremely painful. Furthermore, classical biopsies may be taken from a relatively small area of the tumor and may not be representative of it as a whole^[40]^. Although depending on the neoplasia might be useful to use urine, cerebrospinal fluid, and even saliva as a liquid biopsy, the body fluid that is most useful for this application is blood^[39]^. An additional advantage of using liquid biopsies from blood is that they can be multiplexed and implemented with classical as well as next-generation cancer profiling analyses, including circulating bone turnover biomarkers such as carboxy-terminal telopeptide cross-linked type 1 collagen, pro-peptide of type 1 collagen, bone sialoprotein, tartrate-resistant acid phosphatase 5B (TRAcP5B), and osteoprotegerin^[41,42]^; metabolites such as pyridinoline and deoxypyridinoline^[43]^; and protein markers correlating with poor outcome in bone cancers, including vascular endothelial growth factor (VEGF)^[44]^, metallothionein^[45]^, IL-4, and IL-8^[46,47]^. There are three main types of actionable biological materials that can be obtained from a blood liquid biopsy: (i) circulating tumor cells (CTCs); (ii) cell-free circulating tumor DNA (ctDNA); and (iii) extracellular vesicles (EVs, also referred to as exosomes)^[48]^.

#### CTCs

As the name suggests, CTCs are tumor cells that extravasate into the blood flow. They were first identified by Ashworth more than 150 years ago and are now commonly used in clinical practice^[49]^. Although isolating CTCs has historically been a challenge because of their low number in the general circulation, newly developed microfluidic platforms (e.g., the FDA-approved CellSearch platform) are making this task easier, and next-generation sequencing approaches made it possible to deeply phenotype every single circulating tumor cell isolated^[50]^. These important advances mean that a simple liquid biopsy can provide information about the genetic mosaicism of the primary tumor, their mutational landscape, epigenetics, and even their gene and protein expression.

#### Circulating tumor (ct)DNA

Cell-free ctDNA arises from apoptotic and necrotic primary tumor cells, which release their cellular content into the general circulation, as demonstrated by the fact that most of the ctDNA detected is 180-200 bp in length, which is consistent with what is observed in apoptotic cells^[48]^. Circulating tumor DNA can provide important information about tumor mutations and copy number variation and suggests whether to proceed with a specific-target therapy or not. For example, the V600E mutation in v-raf murine sarcoma viral oncogene homolog B1 (BRAF) is found in several tumors, including metastatic colorectal cancer^[51]^, melanoma^[52,53]^, papillary thyroid cancer^[54]^, etc. The V600E variant of BRAF is targetable with drugs such as vemurafenib, dabrafenib, and trametinib, and its presence can drive the choice of therapy^[55]^. 

#### Extracellular vesicles

The third class of biological material that is usable as liquid biopsy includes extracellular vesicles. These are lipid bilayer particles ranging from 30 to 1000 nm in diameter, which, according to their size, can be classified into three main types: apoptotic bodies, large extracellular vesicles (also known as microvesicles), and small EVs (also known as exosomes). These also differ in biogenesis and biological function^[56,57]^. Importantly, EVs secreted by cells often mimic the molecular composition of the cell of origin, and they contain DNA, miRNAs, mRNAs, proteins, and other biological molecules that are interesting for theranostic and prognostic purposes^[56,58]^. It has been thoroughly demonstrated that EVs have an important role in cancer^[59,60]^, including bone metastases^[60-63]^, osteosarcoma^[64-66]^, and Ewing sarcoma^[67,68]^, and cancer cells secrete more EVs than their normal counterparts^[69]^. Moreover, hypoxia, higher intracellular calcium or lower pH, oxidative stress, ionizing radiation, and ultrasounds have all been shown to increase EV production in both normal and cancer cells^[70]^.

**Figure 1:**
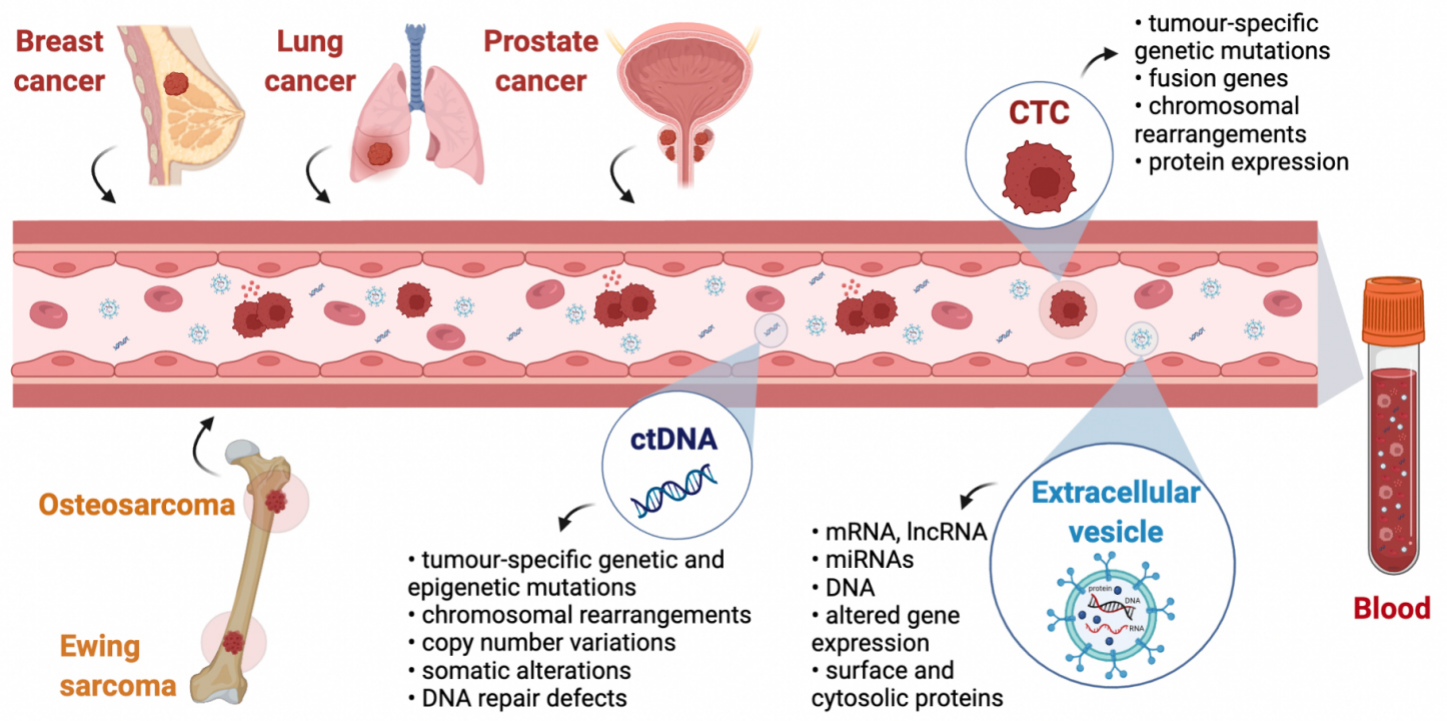
Liquid biopsies in primary and secondary bone cancers. Cell-free ctDNA, CTCs, and EVs are released into the general circulation by both primary (osteosarcoma and Ewing sarcoma) and secondary (breast, lung, and prostate) cancers and can be collected with a simple blood draw. Studying these three cancer-derived components by molecular or cellular analysis is termed liquid biopsy. Much information about the tumor can be achieved by analyzing CTCs, ctDNA, and EVs, making liquid biopsies a minimally invasive alternative to classical biopsies. Created with BioRender.com. ctDNA: Circulating tumor DNA; CTCs: circulating tumor cells; EVs: extracellular vesicles.

EVs are especially interesting for studying RNAs because these molecules are prone to be degraded in the circulation; however, when they are encapsulated into EVs, they become more stable and can be analyzed to gain insights into the transcriptional profile of the cells of origin, as well as regarding miRNAs and long non-coding RNAs^[71]^. A summary of the primary and secondary tumors and the potential information that can be gained by liquid biopsies is schematized in Figure 1. 

Adopting different approaches to acquire all this biological information from a patient’s blood could be possible to perform accurate diagnosis, estimate prognosis, and even monitor the onset of drug-resistant clones to make informed therapeutic choices and change therapy during treatment^[72]^.

Despite the advances in the field at the preclinical level, clinical adoption of bone liquid biopsies remains poor due to costs, scalability, reproducibility, and isolation methods. In the following sections, we focus on the recent developments, as well as preclinical and clinical applications of liquid biopsies in bone oncology, focusing on both secondary [Table 1] and primary [Table 2] tumors.

**Table 1:** Selected studies about liquid biopsy applications in bone metastatic tumors

**Tumor type**	**Source material**	**Target**	**Utility/use**	**References**
Breast cancer	ctDNA	Tumor chromosomal rearrangements, *ESR1* mutations	Early diagnosis of BM	[74,92]
	ctDNA	*TP53*, *PIK3CA*, *ESR1* mutations	Prognosis and treatment efficacy	[89,93]
	ctDNA	*PIK3CA* mutations	Diagnosis of BM	[90]
	ctDNA	Somatic genomic alterations (*PIK3CA* and *TP53*)	Prognosis and treatment efficacy	[91]
	CTCs	Baseline CTC/mL of blood (≥ 5 CTC/7.5 mL)	Diagnosis and prognosis of BM	[80,81,89]
	EVs	Upregulation of *SPP1*, *HSP90AA1*, *IL3*, *VEGFA*, *PTK2* and *YWHAZ* genes	Early detection of BM	[94]
Lung cancer (NSCLC)	ctDNA	*KRAS*, *EGFR*, *BRAF* mutations	Early diagnosis of BM	[77]
	ctDNA	*KRAS* and *EGFR* mutations	Diagnosis and prognosis of BM	[78]
	CTCs	CTCs/mL of blood ​​(≥ 5 CTC/7.5 mL)	Diagnosis and prognosis of BM	[84,85]
	EVs/cmiRNAs	hsa-miR-574-5p, hsa-miR-328-3p, hsa-miR-423-3p	Early detection and monitoring of BM	[95]
Prostate cancer	ctDNA	*TP53* mutations and DNA repair defects	Diagnosis of BM	[79]
	CTCs	CTCs/mL of blood (≥ 5 CTC/7.5 mL)	Diagnosis and prognosis of BM	[86]
	EVs/cmiRNAs	miR-181a-5p	Diagnosis of BM	[96]

NSCLC: Non-small cell lung cancer; ctDNA: cell-free circulating tumor DNA; CTCs: circulating tumor cells; EVs: extracellular vesicles; cmiRNAs: circulating miRNAs; BM: bone metastasis; ESR1: estrogen receptor 1; TP53: tumor protein 53; PIK3CA: phosphatidylinositol-4,5-bisphosphate 3-kinase catalytic subunit alpha; SPP1: secreted phosphoprotein 1; HSP90AA1: heat shock protein 90 alpha family class A member 1; IL3: interleukin-3, VEGFA: vascular endothelial growth factor A; PTK2: protein tyrosine kinase 2; YWHAZ: tyrosine 3-monooxygenase/tryptophan 5-monooxygenase activation protein; BRAF: B-Raf proto-oncogene; serine/threonine kinase; KRAS: KRAS proto-oncogene; EGFR: epidermal growth factor receptor.

**Table 2:** Selected liquid biopsy applications in primary bone tumors

**Tumor type**	**Source material**	**Target**	**Utility/use**	**References**
Osteosarcoma	ctDNA	Somatic mutations	Diagnosis and prognosis	[100]
	ctDNA	Chromosome arm 8q copy number gains	Genotyping and diagnosis	[101]
	CTCs	Baseline CTC/mL of blood (≥ 5 CTC/7.5 mL)	Diagnosis, prognosis, response to therapy	[104,105]
	CTCs	CTC count variations after therapy	Prognosis and treatment efficacy	[106,107]
	EVs	Transcriptomic alterations	Diagnosis of BM	[111]
	EVs/cmiRNAs	miR-148a, -574-3p, -214, -335-5p, -491, -221, -191, -421, -124, -101 and -195	Diagnosis and prognosis	[112]
Ewing sarcoma	ctDNA	*STAG2* and *TP53* mutations, *EWSR1-FLI1* and *EWSR1-ERG* fusion genes	Diagnosis, prognosis and response to therapy	[102,103]
	CTCs	CTCs count, CD99 expression and chromosomal translocations (EWSR1- FL11 fusion gene)	Diagnosis and prognosis of metastasis	[108]
	EVs/cmiRNAs	miR-125b	Diagnosis, prognosis and response to therapy	[121]
	EVs	Proteomic content (CD99, HINT1 and NGFR)	Diagnosis and prognosis	[122]

CtDNA: Cell-free circulating tumor DNA; CTCs: circulating tumor cells; EVs: extracellular vesicles; cmiRNAs: circulating miRNAs; STAG2: stromal antigen 2; TP53: tumor protein 53; EWSR1: Ewing sarcoma RNA binding protein 1; FLI1: friend leukemia integration 1 transcription factor; ERG: erythroblast transformation specific-related gene; HINT1: histidine triad nucleotide-binding protein 1; NGFR: nerve growth factor receptor.

### Liquid biopsy in bone metastasis 

All the key components in liquid biopsies, i.e., ctDNA, CTCs, and EVs, have been exploited to monitor bone metastases secondary to breast, prostate, and lung cancers at the preclinical level.

#### ctDNA for bone metastasis detection

Circulating tumor DNA analysis may provide a complete genetic profile of the mutational landscape of metastatic disease, and is also correlated with patients’ relapse or changes in response to surgical or pharmacological treatment^[73]^. In this regard, a retrospective study of patients with primary breast cancer showed that the detection of metastatic disease, also spread in the bone, was possible by serial measurements of selected tumor-specific chromosomal rearrangements in ctDNA using droplet-based digital PCR technologies from plasma samples, with an average of almost one year before clinical recurrence detection during the follow-up of the disease, and the ctDNA amount was directly proportional to poor survival^[74]^. This highlights the possibility of using ctDNA detection as a diagnostic tool for earlier prediction of metastasis. Liquid biopsies also hold the potential to detect minimal residual disease (MRD), thus providing indications for therapy and prognosis. Detection and analysis of plasma tumor-associated ctDNA were found to be a good indicator of MRD identification and monitoring in breast cancer patients with a high risk of recurrence^[75,76]^. In particular, the detection of ctDNA at baseline was associated with a higher incidence of bone metastasis and subsequent poor prognosis in newly diagnosed patients with advanced non-small cell lung cancer (NSCLC)^[77]^. Consistently, quantification and analysis of ctDNA in late-stage NSCLC patients revealed that higher ctDNA levels were detected in the group of patients with bone metastasis^[78]^. It should be noted that using liquid biopsies as a means of detecting MRD is still a developing field, and the risk of false positives and false negatives is a concrete one that needs to be addressed in larger-scale longitudinal studies. Regarding the use of ctDNA, to the best of our knowledge, there are no studies focusing specifically on prostate cancer bone metastases. However, Vandekerkhove *et al.* showed that prostate cancer patients with visceral metastases had higher levels of ctDNA than bone metastatic patients^[79]^.

#### CTCs for bone metastasis detection

As for CTCs, they have also been proven useful for diagnosis, prognosis, and monitoring treatment efficacy in metastatic breast cancer. Indeed, CTC quantification and characterization in patients with metastatic breast cancer have been associated with the presence of bone and liver metastases^[80]^. 

Remarkably, higher CTC counts correlated with multiple metastatic sites, while a lower CTC count was found in bone-only metastatic breast cancer patients, who also presented with a better prognosis^[81,82]^, indicating that patients with less advanced disease had fewer CTCs. Moreover, patients with only one or two bone metastases had sharply fewer CTCs compared to patients with more bone metastases^[82]^. CTCs seem to have a subpopulation of metastasis-initiating cells that express epithelial cellular adhesion molecule, CD44, CD47, and c-MET. Once these cells were sorted and transplanted from a patient to immunocompromised mice, they induced bone, lung, and liver metastases^[83]^.

CTC detection and quantification have shown prognostic potential in lung cancer patients, especially in advanced NSCLC, the most common histological subtype, highly metastasizing to bone. In the last decade, some studies showed that a high number of CTCs is a predictive and prognostic indicator of bone metastasis^[84,85]^ in advanced lung cancer patients. The prognostic utility of CTCs in monitoring prostate cancer bone metastases was validated by multiple prospective studies performed on peripheral blood of metastatic castration-resistant prostate cancer patients after treatment. It was found that CTC counts ≥ 5 per 7.5 mL of blood are associated with lower overall survival and are predictive of bone metastases^[86-88]^.

Some studies suggested a liquid biopsy approach involving simultaneous detection and quantification of both CTCs and ctDNA. A valid example to report is the COMET (NCT01745757) prospective study, conducted on peripheral blood samples collected before and after chemotherapy from a homogeneous group of HER2-negative breast cancer patients. In patients with bone, liver, and brain metastasis, CTCs were greater in number and ctDNA analyses revealed at least one mutation in tumor protein 53, phosphatidylinositol-4,5-bisphosphate 3-kinase catalytic subunit alpha, and estrogen receptor 1 (*ESR1*) genes compared to non-metastatic patients^[89]^. These tumor-specific mutations in ctDNA analysis from plasma of patients with visceral and non-visceral metastasis, including bone, were confirmed in other studies^[90-93]^, making this CTC-ctDNA signature potentially useful for diagnosis and prognosis of metastatic breast cancer. 

#### Extracellular vesicles cargo and miRNAs for bone metastasis detection

As for EVs, starting from a meta-analysis conducted on publicly available microarray datasets derived from multiple-cancer type patients with and without bone metastasis, Bhadresha and colleagues identified 15 genes that were consistently upregulated in bone metastatic patients, and then validated their expression in patient serum-derived EVs. Among these, five were confirmed upregulated by qPCR in EVs isolated from the serum of breast and lung cancer patients harboring bone metastasis, namely, heat shock protein 90 alpha family class A member 1 (HSP90AA1), secreted phosphoprotein 1 (also referred to as osteopontin), IL-3, VEGFA, and protein tyrosine kinase 2. The authors concluded that this EV-derived mRNA gene signature could be a useful predictive tool for the early detection of bone metastases in breast and lung cancers^[94]^. Few studies focused their attention on EV-derived miRNAs released from lung cancer cells. A retrospective study was conducted on plasma-derived EV miRNAs from NSCLC patients, focusing on their potential as biomarkers of early detection and monitoring of bone metastasis. In particular, hsa-miR-574-5p, hsa-miR-328-3p, and hsa-miR-423-3p have been suggested as potential biomarkers for bone metastasis^[95]^. As for prostate cancer, EV-derived miR-181a-5p- was recently found upregulated in bone-metastatic prostate patients by Wang *et al.* and proposed as a biomarker for its diagnosis; similar findings were shown by Bryant *et al.* for miR-141 and miR-375^[96,97]^. Additionally, prostate microparticles, a type of prostate-specific EVs, were found to be more numerous in metastatic prostate cancer compared to non-metastatic cancer and showed better predictive ability than CTCs detected with the FDA-approved CellSearch system^[98]^. It is worth mentioning that a platform based on urine exosomal gene expression, termed ExoDX, was recently developed and tested in a utility trial on more than 500 patients. ExoDX-based scoring could predict high-grade prostate cancer in patients with uncertain prostate-specific antigen scores better than the gold standard (ROC AUC 0.7 *vs.* 0.62) and, conversely, it managed to predict which patients only had benign prostate hyperplasia, thus avoiding unnecessary biopsies^[99]^.

### Liquid biopsy in primary bone malignancies 

#### ctDNA detection 

Few studies have been performed on plasma-derived ctDNAs analysis from osteosarcoma patients^[100,101]^. One study focused on somatic mutations associated with tumor burden and disease outcome. Here, the researchers, using the targeted next-generation sequencing (NGS) approach, identified tumor-specific somatic alterations by comparing tumor and germline DNA extracted from peripheral mononuclear blood cells with tissue biopsies and demonstrated that patient-specific somatic alterations can be detected in ctDNA collected from the plasma samples at various stages of treatment, which allows the monitoring of disease burden^[100]^. In another study, Shulman and colleagues showed that ctDNA levels detected by NGS hybrid capture assay in peripheral blood samples of patients with newly diagnosed localized osteosarcoma and Ewing sarcoma may be associated with tumor burden, relapse, and negative disease outcomes. Interestingly, ctDNA analysis led to the identification of novel genomic features of osteosarcoma, including chromosome arm 8q copy number gains^[101]^. Specific and well-characterized genetic mutations, such as stromal antigen 2 (STAG2) and TP53 loss-of-function mutations, translocation events, and fusion genes [Most commonly, EWSR1-FLI1 and EWSR1-erythroblast transformation specific-related gene (ERG)], have been found expressed in Ewing sarcoma patients, leading to the opportunity to monitor this bone malignancy through ctDNA^[102]^. The above-described retrospective study conducted by Shulman and colleagues showed an association between ctDNA detection in plasma samples and a poor clinical outcome in patients with newly diagnosed Ewing sarcoma and identified from ctDNA analysis additional genomic information, such as EWSR1 fusion and STAG2 loss-of-function mutations^[101]^. Another promising application for liquid biopsy in Ewing sarcoma was provided by Hayashi *et al.*, who found that tumor burden and response to therapy were related to increased levels of circulating *EWSR1-FLI1 *fusion gene in plasma of patients. Moreover, they observed that *EWS-FLI1 *levels in the circulation decreased after chemotherapy or surgery and then started to rise again during tumor recurrence^[103]^.

#### CTCs analysis

CTCs have been proposed as potential predictive and prognostic markers for osteosarcoma metastasis^[104]^. A prospective study undertaken by Li *et al.* revealed a higher number of CTCs detected at baseline in peripheral blood of metastatic osteosarcoma patients compared to ones with localized disease. Moreover, they observed that CTC count was inversely correlated with the patient response after neoadjuvant chemotherapy^[105]^. Consistently, other preclinical studies have shown that CTC count variations after therapy or surgical resection can reflect the tumor’s sensitivity to the treatment and may be a good indicator of metastasis^[106,107]^, highlighting the clinical interest in dynamic monitoring of CTC changes for understanding treatment efficacy and detecting disease recurrence or metastasis in time. In particular, the presence of an increased percentage of CTCs with mesenchymal phenotype (identified by the epithelial-to-mesenchymal transition markers) in peripheral blood of a small group of osteosarcoma patients after chemotherapy treatment was associated with reduced disease-free survival, leading to the possibility to predict disease relapse and lung metastasis occurrence^[106,107]^.

A few studies have reported the use of tumor-specific makers for CTC isolation and characterization in Ewing sarcoma patients, including CD99 expression and presence of chromosomal translocations, such as amplification of EWSR1-FLI1 transcript fusion gene, by using different methods^[108,109]^. Others have suggested that detection of CTCs at diagnosis in Ewing sarcoma patients may be associated with worse clinical outcomes and increased risk of recurrent disease or development of overt metastasis^[109,110]^.

#### Extracellular vesicles cargo and miRNAs

EVs have recently been studied as diagnostic or prognostic serum biomarkers via a liquid biopsy approach in osteosarcoma. Circulating EVs RNA profiling of metastatic *vs.* primary osteosarcoma samples allowed the detection of multiple transcriptomic alterations in the former, providing a new clinically relevant approach to track metastatic osteosarcoma^[111]^.

Several miRNAs, which are known to at least partially circulate inside EVs, with oncogenic or antitumor-suppressor roles in osteosarcoma, have been detected in the peripheral blood of patients. Some of them are emerging as important diagnostic and prognostic biomarkers, such as miR-148a^[112]^, miR-574-3p, miR-214 and miR-335-5p^[113]^, miR-49^1^^[114]^, miR-221^[115]^, miR-191^[116]^, and miR-421^[117]^. Conversely, miR-124^[118]^, miR-101^[119]^, and miR-195^[120]^ were shown to be downregulated in the serum of osteosarcoma patients, compared to healthy individuals. A potential application for these findings could be to establish a predictive strategy for osteosarcoma prognosis using a combination of these miRNAs. Circulating miRNAs have also recently become the subject of study in Ewing sarcoma. As an example, a widely studied circulating miRNA related to Ewing sarcoma progression is miR-125b, which was found decreased in patients serum after surgical resection when compared to healthy controls^[121]^. In the same study, its downregulation in the group of patients analyzed was also correlated with poor response to chemotherapy^[121]^. Recently, the research focus is shifting towards Ewing sarcoma-derived EV cargo as a prognostic biomarker source, particularly to their protein content. Samuel *et al.* identified and used CD99, histidine triad nucleotide-binding protein 1 (HINT1), and nerve growth factor receptor (NGFR) membrane proteins as potential biomarkers of Ewing sarcoma-derived small EVs. They developed an approach of immuno-enrichment of Ewing sarcoma-associated small EVs based on these EV surface proteins, for the subsequent detection of EWS-FLI1 and EWS-ERG fusion transcripts in EVs isolated from plasma of both localized and metastatic patients^[122]^.

### CLINICAL IMPLICATION OF LIQUID BIOPSY IN MONITORING DRUG RESISTANCE

### Liquid biopsies in chemoresistance of primary and secondary bone cancers: an overview

A growing body of evidence suggests that the tumor secretome, including circulating-free DNA fragments from drug-resistant cells containing tumor-specific genetic and epigenetic mutations, is highly abundant in plasma; thus, the role of blood-based liquid biopsy is fundamental for this field of research^[123]^. Although several studies have been conducted on plasma samples of relatively small cohorts of patients, trying to identify resistance mutations that occur during treatment, the data obtained thus far are clinically informative about therapy response, but still not completely validated in clinical practice. According to the different types of tumors, quantification and analysis of ctDNA were found to be usable as a viable tool for this purpose^[124]^.

A good example suggesting that ctDNA is a valuable strategy to monitor treatment efficacy was provided by Schiavon *et al.*, who showed that ESR1 mutations found in ctDNA from plasma of metastatic breast cancer patients previously treated with aromatase inhibitors are associated with resistance to endocrine therapy and shorter progression-free survival^[125]^. Additionally, liquid biopsy has also proved to be useful for the identification of biomarkers associated with cyclin-dependent kinase inhibitors (CDKi) resistance and for predicting the subsequent development of metastatic disease, in hormone receptor-positive/human epidermal growth factor receptor 2 negative (HR+/HER2-) advanced breast cancer patients. Patients treated with CDKi in combination with endocrine therapy presented with specific therapy-induced mutations in the ctDNA analyzed, including retinoblastoma*, ESR1, *fibroblast growth factor receptor 1, or phosphatidylinositol-4,5-bisphosphate 3-kinase catalytic subunit alpha alterations^[126-128]^, which could be useful for predicting disease outcomes and drive therapeutic decisions. It is also important to mention the detection and quantification of CTC acquired resistance, which may also act as a prognostic marker able to predict treatment outcomes^[129]^. Similarly, in castration-resistant prostate cancer patients treated with docetaxel, CTC count in blood was found to be an effective indicator of treatment sensitivity and patient survival^[130]^.

Due to its high genetic instability, osteosarcoma treatment is often hampered by the acquisition of chemoresistance following therapy-induced selective pressure. Some studies observed that low serum levels of miR-375 in osteosarcoma patients were related to poor tumor response to preoperative chemotherapy^[131]^. Recently, other tumor-associated miRNAs have been linked with osteosarcoma chemoresistance, such as miR-491, which was found to be decreased in serum from osteosarcoma patients compared with healthy control subjects, and this decreased serum miR-491 level is correlated with increased metastasis, poor chemoresponse, and lower survival rate^[114]^. In contrast, the serum levels of miR-21 were found to be significantly higher in patients with osteosarcoma than in control subjects and correlated with advanced Enneking stage and chemotherapeutic resistance^[132]^.

As for the development of chemoresistance in Ewing sarcoma, a recent study showed that circulating miR-125b levels were decreased in patient serum, and this was associated with poor response to chemotherapy^[121]^.

### Implication of extracellular vesicles in chemoresistance

Extracellular vesicles are emerging as key players in the transfer of drug resistance^[133]^; hence, they could be useful for monitoring the onset of this phenomenon during treatment.

Among the most studied constituents of tumor-derived EVs cargo, miRNAs have been identified as potential biomarkers for monitoring chemoresistance. EVs from doxorubicin-resistant osteosarcoma MG63 cells express high levels of the membrane transporter pump P-glycoprotein, transferring resistance to doxorubicin treatment to sensitive cancer cells horizontally^[134]^. In another report, miR-25-3p upregulation in the blood of osteosarcoma patients correlated with increased tumor growth and drug resistance^[135]^.

 Wei and colleagues demonstrated the role of EV-derived miR-222-3p detected in the serum of NSCLC patients for predicting gemcitabine sensitivity and identifying patients with the aggressive advanced and resistant disease^[136]^. In addition, higher levels of EV-derived miR-425-3p were found in the serum of NSCLC platinum-resistant patients compared with platinum-sensitive patients^[137]^.

In breast cancer, it was found that EV-shuttled miR-222 released by doxorubicin-resistant breast cancer cells was locally transferred to M2 macrophages, thus activating their polarization. In these cells, overexpression of miR-222 suppressed phosphatase and tensin homolog gene, resulting in phosphorylation of Akt and activation of Akt signaling, which in turn supports cancer cells proliferation, migration, and invasion in a positive feedback loop. Accordingly, increased levels of miR-222 were found in EVs from plasma of patients presenting with chemoresistant breast cancer^[138]^. In another study, it has been demonstrated that the human osteotropic breast cancer cell line MDA-MB-231 treated with paclitaxel was able to release EVs enriched in the cell survival protein Survivin^[139]^.

Interestingly, EVs can also act directly against anti-neoplastic agents. In fact, a recent study showed that HER2-positive breast cancer-derived EVs interfere with the activity of trastuzumab, acting as decoy receptors for it^[140]^. In fact, EVs secreted by those cancer cells were found to have HER2 on their surface, and trastuzumab administered systemically binds it, hence making the amount of antibody available to bind cells lower. Furthermore, higher levels of glutathione S-transferase P1 (GSTP1) mRNA were found by Yang *et al.* in EVs from the serum of non-responding breast cancer patients treated with neoadjuvant chemotherapy compared to the responders. Intriguingly, they also demonstrated that GSTP1-containing EVs transferred drug resistance horizontally, and hence proposed their use as negative predictive factors of chemoresistance and clinical outcomes in breast cancer patients treated with anthracycline/taxane-based therapy^[141]^. Similar results were observed for EV-bound transient receptor potential channel 5 mRNA found in peripheral blood of metastatic breast cancer patients, which could be a potential predictive marker of chemoresistance^[142]^.

Kharaziha *et al.* conducted proteomic analysis on EVs derived from prostate cancer cells sensitive *vs.* resistant to docetaxel, identifying multidrug resistance protein 1 (MDR-1), MDR-3, endophilin-A2, and poly(A) binding protein 4 as proteins enriched in the latter as well as present in EVs from the serum of castration-resistant prostate cancer patients, suggesting that EVs may be used as biomarker candidates for predicting therapeutic response or resistance^[143]^.

Larger longitudinal studies will be crucial to validate the biomarkers identified thus far, but the field holds great promise.

### Factors hindering the clinical application of liquid biopsies

Although liquid biopsies are extremely promising tools, there are issues that need to be addressed before widespread clinical adoption can occur. First, since the techniques used are extremely sensitive, even small differences at the sample collection or processing level can cause significant differences in the final outcome. The use of serum instead of plasma, for example, can increase the amount of cell-free DNA released from other sources such as leukocytes, thus reducing the diagnostic ability of NGS-based assays, especially when trying to detect rare variants^[144]^. Moreover, lifestyle-related factors can affect the release of cell-free DNA in the general circulation, thus constituting an array of possible confounding factors that are hard to identify and characterize systematically^[145]^. As for CTCs, they are usually extremely rare and hard to capture, and although the CellSearch method has provided the field with a standardized method, CTCs captured this way are not viable. This means they can only be used for DNA and FACS/Immunofluorescence studies, but not for RNA-based assays or functional assays, including patient-derived xenografts or *in vitro* drug sensitivity tests^[146,147]^. Moreover, CTC analysis has some of the drawbacks as classical biopsies, since they are not necessarily representative of the entire tumor, but only a subpopulation of cells that were able to migrate and survive in the circulation. A possible solution under investigation to at least partially solve this issue is choosing different sites for the blood collection. It has been reported that arterial blood and blood withdrawn from a closer site to the primary tumor may provide a higher number of CTCs^[148,149]^. Significant efforts have been made by societies in both the United States and Europe to draw standard guidelines for preanalytical sample treatment, but while the consensus is widely accepted, liquid biopsies remain technically challenging for both the clinician and the analytical lab staff, which need specific training and facilities that are not always available locally, as well as training to interpret the results correctly^[150]^. In addition to the general problems outlined above, EVs carry their own challenges at the preanalytical level. They are the most recently recognized source of biological information, and therefore their development as liquid biopsy tool is still at an early stage. A key example is the issue of EV isolation. There are several methods that are currently available to isolate EVs, namely differential ultracentrifugation, isopycnic ultracentrifugation, size-exclusion chromatography, polymer-based precipitation, immune-capture, asymmetric field flow fractionation, ultrafiltration, and the countless possible combinations among them^[151]^. Unfortunately, no technique is absolutely superior to another, and, depending on the one investigator’s use for EV isolation, the results obtained may vary^[151]^. Moreover, as stated above, EV secretion is stimulated by several factors that may relate to one’s lifestyle, which may make the detection of tumor-specific exosomes challenging^[70]^. Finally, while these considerations are valid for oncology in general, the field of bone oncology is currently lacking specific clinical trials, although we are confident that it is just a matter of time before this gap is filled.

## DISCUSSION AND CONCLUSIONS

Liquid biopsies have the potential to become one of the most powerful instruments in the clinical oncologist’s toolkit, making diagnosis and prognosis increasingly accurate and therapy more personalized. In fact, the technique is rapidly gaining popularity, albeit still with somewhat limited success, as happens with most new implementations at early stages^[39]^. This is particularly important in bone oncology, where minimally invasive techniques to evaluate clinical response to therapy and prognosis are limited. Despite this, the field is currently lagging behind, and there is a significant gap that needs to be filled before concretely applying liquid biopsies in clinical practice. The potential benefits of liquid biopsies not only are related to the life quality and expectancy of cancer patients but also extend to the cost-effectiveness of treatments. In this regard, a cost-consequence analysis was recently conducted comparing the use of tissue biopsy alone *vs.* tissue biopsy-liquid biopsy combined diagnostic strategy, in NSCLC^[152]^, where the application of the latter implied lower overall medical expenses for the healthcare system compared to tissue biopsy alone. Additionally, liquid biopsies not only are a potential means of diagnosis but also can be used as risk-profiling tools, which could be able to identify subjects that are at risk for bone metastases, so that preventive therapies can be initiated. Of course, CTCs have been the most widely used in preclinical and clinical practice thus far, gaining FDA approval for use in some metastatic tumor prognosis (i.e., colorectal, breast, and prostate cancer), mainly due to the development of well-standardized isolation and analytical techniques. Nevertheless, ctDNA and tumor-derived EVs may become even more important than CTCs in the future, especially for personalized medicine. Indeed, they are easier to detect and characterize and can genetically reflect the tumor as a whole, providing the potential for real-time monitoring of tumor progression and development of chemoresistance, as has also been proposed or demonstrated in other neoplasias^[153-155]^. Liquid biopsies could also be useful in tracking metastases before they become overt and therapy resistance by implementing them in standard follow-up protocols and analyzing the emergence of mutations that are important for therapy resistance and metastasis, as the pioneering work by the Bardelli group already demonstrated in colorectal carcinoma, although timelines may need to be readapted^[153,154]^. Moreover, EVs also play an active role in malignancy and chemoresistance, and targeting them could be a valuable tool in the oncologist’s toolkit, especially considering that some of the most commonly used EV secretion inhibitors are clinically approved for other conditions (manumycin A, D-pantethine, imipramine, tipifarnib, neticonazole, climbazole, ketoconazole, and triademenol)^[156]^ and could be repurposed to reduce EV-induced chemoresistance. We believe this research area is largely underexplored and will see an increase of interest in the next few years, also considering the advances in next-generation sequencing that could eventually lead to a “single-EV sequencing”, which would really open a new avenue for the field^[157]^.

## DECLARATIONS

### Copyright

© The Author(s) 2022.
